# Signature selection forces and evolutionary divergence of immune-survival genes compared between two important shrimp species

**DOI:** 10.1371/journal.pone.0280250

**Published:** 2023-01-12

**Authors:** Tze Chiew Christie Soo, Subha Bhassu

**Affiliations:** 1 Department of Genetics and Molecular Biology, Animal Genetics and Genome Evolutionary Laboratory (AGAGEL), Institute of Biological Sciences, Faculty of Science, University of Malaya, Kuala Lumpur, Malaysia; 2 Terra Aqua Laboratory, Centre for Research in Biotechnology for Agriculture (CEBAR), Research Management and Innovation Complex, University of Malaya, Kuala Lumpur, Malaysia; Chang Gung University, TAIWAN

## Abstract

In recent years, shrimp aquaculture industry had grown significantly to become the major source of global shrimp production. Despite that, shrimp aquaculture production was impeded by various shrimp diseases over the past decades. Interestingly, different shrimp species demonstrated variable levels of immune strength and survival (immune-survival) ability towards different diseases, especially the much stronger immune-survival ability shown by the ancient shrimp species, *Macrobrachium rosenbergii* compared to other shrimp species. In this study, two important shrimp species, *M*. *rosenbergii* and *Penaeus monodon* (disease tolerant strain) (uninfected control and *Vp*_AHPND_-infected) were compared to uncover the potential underlying genetic factors. The shrimp species were sampled, followed by RNA extraction and cDNA conversion. Five important immune-survival genes (C-type Lectin, HMGB, STAT, ALF3, and ATPase 8/6) were selected for PCR, sequencing, and subsequent genetics analysis. The overall genetic analyses conducted, including Analysis of Molecular Variance (AMOVA) and population differentiation, showed significant genetic differentiation (p<0.05) between different genes of *M*. *rosenbergii* and *P*. *monodon*. There was greater genetic divergence identified between HMGB subgroups of *P*. *monodon* (uninfected control and *Vp*_AHPND_-infected) compared to other genes. Besides that, based on neutrality tests conducted, purifying selection was determined to be the main evolutionary driving force of *M*. *rosenbergii* and *P*. *monodon* with stronger purifying selection exhibited in *M*. *rosenbergii* genes. Potential balancing selection was identified for *Vp*_AHPND_-infected HMGB subgroup whereas directional selection was detected for HMGB (both species) and ATPase 8/6 (only *P*. *monodon*) genes. The divergence times between *M*. *rosenbergii* and *P*. *monodon* genes were estimated through Bayesian molecular clock analysis, which were 438.6 mya (C-type Lectin), 1885.4 mya (HMGB), 432.6 mya (STAT), 448.1 mya (ALF3), and 426.4 mya (ATPase 8/6) respectively. In conclusion, important selection forces and evolutionary divergence information of immune-survival genes between *M*. *rosenbergii* and *P*. *monodon* were successfully identified.

## 1 Introduction

The shrimp aquaculture industry is an essential global economic sector, especially for some low- and middle-level developing economies in Latin America and Asia [1, 2]. The main cultured shrimp species, including *Litopenaeus vannamei*, *Penaeus monodon*, and *Macrobrachium rosenbergii* had contributed to around 6.0 million tonnes of the annual global shrimp aquaculture production in the year 2018 [2]. However, shrimp aquaculture production had been heavily impeded by different shrimp diseases, which was also associated with high economic losses [3]. There was a previous reporting of an estimated annual production loss of 22% and corresponding value loss of US$ 1 billion due to shrimp diseases [4]. Common shrimp diseases include Acute Hepatopancreatic Necrosis Disease (AHPND), White Spot Disease (WSD), Hepatopancreatic Microsporidiosis, Yellow Head Disease, and Infectious Myonecrosis [5].

AHPND is a well-known shrimp bacterial disease that can lead to 40–100% mortality within the early 35 days after shrimp stocking [6]. This shrimp disease had originally emerged from China in the year 2009 and then spread to Southeast Asia and Mexico regions [7, 8]. AHPND disease is mainly caused by a pathogenic strain of Gram-negative *Vibrio parahaemolyticus* bacteria, known as *Vp*_AHPND_ [9, 10]. The *Vp*_AHPND_ bacteria contain a 70-kbp plasmid (pVA1), which can encode for *Photorhabdus* insect-related (Pir) toxins, PirA and PirB [9]. The detection of the AHPND disease can be done through the observation of gross clinical signs, which include pale and atrophied hepatopancreas, empty gut, empty stomach, lethargy, and slow growth [6].

Additionally, differing levels of disease resistance or tolerance were exhibited by different shrimp species towards a particular shrimp disease. The AHPND-susceptible shrimp species are *P*. *monodon*, *Fenneropenaeus chinensis*, and *L*. *vannamei* [11]. Intriguingly, despite being susceptible to Vibriosis disease caused by *V*. *parahaemolyticus* bacteria [12], *M*. *rosenbergii* was shown to be not susceptible to AHPND infection under salinity condition of 20 ppt or lower [13]. Another suitable example would be WSD which caused lethality in penaeid shrimp species, including *P*. *monodon*, *F*. *chinensis*, and *L*. *vannamei* [14]. On the other hand, *M*. *rosenbergii* showed resistance and viral clearance ability towards WSD [15].

Different strategies have been applied to reduce shrimp disease outbreaks, such as selective breeding [16, 17], usage of Specific Pathogen Free shrimps [16, 18], trained immunity [19], biological methods (prebiotics, probiotics, phytobiotics, synbiotics, bacteriophages, and postbiotics) [20], and farm biosecurity management [16]. Selective breeding is crucial for the development of disease resistant or tolerant shrimps, such as selectively bred Taura Syndrome Virus-resistant *L*. *vannamei* shrimps [21] and selectively bred Gill-Associated Virus-tolerant *P*. *monodon* shrimps [22]. Furthermore, selective breeding for disease resistance or tolerance leads to stronger shrimp immune response, as shown by increased immune parameters of selectively bred WSD-resistant *L*. *vannamei* shrimps [23].

The immune genetic differences or alterations that contribute to varying levels of disease resistance or tolerance between different shrimp species or between selectively bred and normal shrimp lines need to be better understood to further improve disease control efforts. Genetic divergence is often caused by selection forces, for example, positive selection or purifying selection, which then leads to genome evolution and enhanced immune response [24, 25]. A greater insight into the vital evolutionary events that happened can be obtained through immune gene divergence time estimations, particularly along the ancient ancestral lines [26, 27].

Shrimps mainly utilize their innate immunity for survival and elimination of invading pathogens. The shrimp innate immune system generally consists of pathogen recognition receptors (C-type Lectin) [28], damage-associated molecular patterns [high mobility group box (HMGB)] [29], signalling pathways [janus kinase (JAK)-signal transducer and activator of transcription (STAT) pathway] [30], antimicrobial peptides [antilipopolysaccharide factor (ALF)] [28], and other immune-related genes (ATPase 8/6). Other important shrimp innate immune responses include prophenoloxidase system, clotting cascade, nodule formation, phagocytosis, encapsulation, and apoptosis [28, 31].

Therefore, in this study, we aim to investigate the important immune genetic differences between disease tolerant *P*. *monodon* and *M*. *rosenbergii* shrimps. The experimental focus was given to five selected vital immune genes, including C-type Lectin, HMGB, STAT, ALF3, and ATPase 8/6. Each of these genes has crucial function in the shrimp innate immune response and survival. Genetic sequences were then obtained for these immune-survival genes and subsequently used for evolutionary genetics analyses between *P*. *monodon* and *M*. *rosenbergii*. The signature genetic divergence, selection forces, and divergence times were successfully determined.

## 2 Materials and methods

### 2.1 Shrimp sample preparations

*Macrobrachium rosenbergii* juvenile prawns (15–20 cm body length) were acquired from a local prawn farm. A total number of 50 prawns were dissected with the hepatopancreas samples collected and stored for subsequent downstream applications. On the other hand, similarly, disease-tolerant crossbred (13^th^ generation Madagascar strain crossed with 5^th^ generation local strain) *Penaeus monodon* juvenile shrimps (15–20 cm body length) were obtained from a local shrimp farm. The shrimps were then kept aseptically for seven days acclimatization (27 shrimps in each tank) in aerated artificial seawater (30 ppt) at 28 ± 1.0°C.

### 2.2 *Vp*_AHPND_ experimental challenge

#### 2.2.1 Pathogen preparations

*P*. *monodon* shrimps suspected with AHPND outbreaks were acquired and validated through gross clinical sign observation and AP3 PCR detection method [32]. The selective propagation of *Vp*_AHPND_ bacteria [33] was then done using digestive organs of *Vp*_AHPND_-infected shrimps through a series of incubations in tryptic soy broth (TSB+), thiosulfate citrate bile salt (TCBS) agar, and tryptic soy agar (TSA+). The propagated bacteria were stored in cryovials (CRYOBANK™) at -80°C for usage in downstream experiments.

#### 2.2.2 Pre-challenge preparations and experimental challenge

The acclimatized *P*. *monodon* shrimps were negatively screened using AP3 PCR method [32] for validation to be *Vp*_AHPND_-free prior to the experimental challenge. During the *Vp*_AHPND_ experimental challenge, the *P*. *monodon* shrimps were infected with *Vp*_AHPND_ bacteria (KS17.S5-1 positive strain) (2 × 10^6^ cfu/ml) [34] using a modified immersion method [10]. The negative control group shrimps were added with sterile TSB+ broth instead of *Vp*_AHPND_ bacteria. The shrimp hepatopancreas samples were obtained at 0, 3, 6, 12, 24, 36, and 48 hours post-infection (hpi) and stored at -80°C. The experimental challenge details were also described in a previous publication [33].

All shrimp tanks were equipped with water filters and aerators. The experimental challenge involved three biological replicates for each treatment and control group. The challenged *P*. *monodon* shrimps were positively screened using AP3 PCR method [32] to confirm the presence of *Vp*_AHPND_ bacteria. The ethical approval was given by University of Malaya for the study within its facilities (Ethical Application Ref: S/31012019/26112018-05/R).

### 2.3 Total RNA extraction and first strand cDNA synthesis

Total RNA samples were extracted from *M*. *rosenbergii* prawn and *P*. *monodon* shrimp hepatopancreas using TransZol Up Plus RNA Kit (TransGen Biotech, Beijing, China). The extracted RNA samples were then used for first-strand cDNA synthesis and DNA contaminant removal using TransScript® One-Step gDNA Removal and cDNA Synthesis SuperMix (TransGen Biotech, Beijing, China). The manufacturer’s protocols were followed for the kits used. The cDNA samples acquired were stored and utilized in downstream experiments.

### 2.4 PCR and sequencing

Primer design was conducted using Primer Quest Tool software (https://sg.idtdna.com/Primerquest/home/Index) by referring to nucleotide sequences either downloaded from NCBI database or selected from previously acquired RNA-Seq analysis data (Differentially Expressed Genes sequences). The detailed information of designed and used primers were provided in S1A & S1B Table. The cDNA sequences of selected immune genes (C-type Lectin, HMGB, STAT, ALF3, ATPase 8/6) were determined through PCR amplification and Sanger Sequencing. A range of 30 to 50 individual sequences were obtained for each of the immune genes for both *M*. *rosenbergii* and *P*. *monodon*. Major regions of the gene sequences (>70%) particularly conserved domains were amplified using the designed primers to ensure validity and importance of the sequences obtained.

The PCR experiments involved the usage of GoTaq® Flexi DNA Polymerase (Promega, Wisconsin, USA) and Eppendorf Mastercycler EP Gradient S (Eppendorf, Hamburg, Germany)/Bio-Rad C1000 Thermal Cycler (Bio-Rad Laboratories, California, USA) instrument. Each PCR reaction had a total volume of 25 μL, including 5.0 μL 5 × GoTaq® Flexi Buffer, 1.5 μL 25 mM MgCl_2_ solution, 0.5 μL 10 mM dNTPs, 0.25 μL GoTaq® DNA Polymerase (5U/μL), 400 nM forward primer, 400 nM reverse primer, 1.2 μL template cDNA, and Nuclease-free water. The PCR amplification condition applied was initial denaturation of 95°C for 5 min, 40 cycles of 95°C for 45 s, T_A_ °C for 45 s and 72°C for 1 min, and final extension of 72°C for 5 min.

Subsequently, the PCR product validation was done through agarose gel electrophoresis analysis, which involved Vivantis 100 bp DNA Ladder/100 bp Plus DNA Ladder (Vivantis Technologies, Selangor, Malaysia) and Major Science MP-300V/MBE-150 device (Major Science, California, USA). The validated PCR products were utilized for Sanger Sequencing analysis. The sequences obtained were then trimmed and compiled using MEGA7 [35] and Chromas (https://technelysium.com.au/wp/chromas/) software. The compiled sequences were used in subsequent genetics analyses.

### 2.5 Evolutionary genetics analysis

The acquired cDNA sequences were compared and analysed through different software, including Arlequin (Version 3.1) [36], DnaSP (Version 5.0) [37], MEGA7 [35], and R [38]. The sequence comparison was focused on different gene sequences of *M*. *rosenbergii* and *P*. *monodon* and different subgroup sequences of uninfected control and *Vp*_AHPND_-infected *P*. *monodon*. The genetics analyses carried out using Arlequin software [36] are Analysis of Molecular Variance (AMOVA), Population Matrix Differentiation, Population Pairwise FSTs, and Neutrality tests (Ewens-Watterson, Tajima’s D, and Fu’s Fs).

Besides that, DNA divergence analysis was done through DnaSP software [37]. The number of conserved or diverged sites were also determined at both nucleotide and amino acid levels through MEGA7 software [35]. Subsequently, genetic differentiation display and PCA analysis were plotted using R software [38] for total cDNA sequences comparison (based on genetic distances). The software packages applied were ape, reshape2, and ggplot2 (genetic differentiation display) and ape, ggfortify, and ggplot2 (PCA analysis) respectively.

### 2.6 Phylogenetic and Bayesian molecular clock dating analyses

For further genetic divergence analysis, the acquired cDNA sequences were checked with JModelTest2 software [39, 40] to identify the optimal models of nucleotide substitution. The optimal nucleotide models were determined based on the best delta values of Akaike and Bayesian information criteria (AIC and BIC) for phylogenetic analysis and Bayesian analysis respectively. The total cDNA sequences were utilized for phylogenetic analysis using MEGAX software [41] through Maximum likelihood method (Number of bootstraps: 1000). Besides that, Bayesian Molecular Clock Dating analysis was also conducted using BEAST2 software [42] with relaxed clock model [43] (Chain length: 10000000 or before infinity and ESS>200) in this study. Different cDNA gene sequences including C-type Lectin, HMGB, STAT, ALF3, and ATPase 8/6 were involved. The genetic divergence time estimations were done by referring to a previously constructed time tree involving crabs, lobsters, and shrimps [44]. The estimated divergence time information between *M*. *rosenbergii* prawn family, Palaemonidae and *P*. *monodon* shrimp family, Penaeidae was utilized as the reference for the genetic divergence time estimation in this study. The prediction of divergence time, ancient divergence period, and evolutionary event was achieved through the Bayesian analysis.

## 3 Results

### 3.1 Genetic conservation or divergence analysis

**Fig 1:**
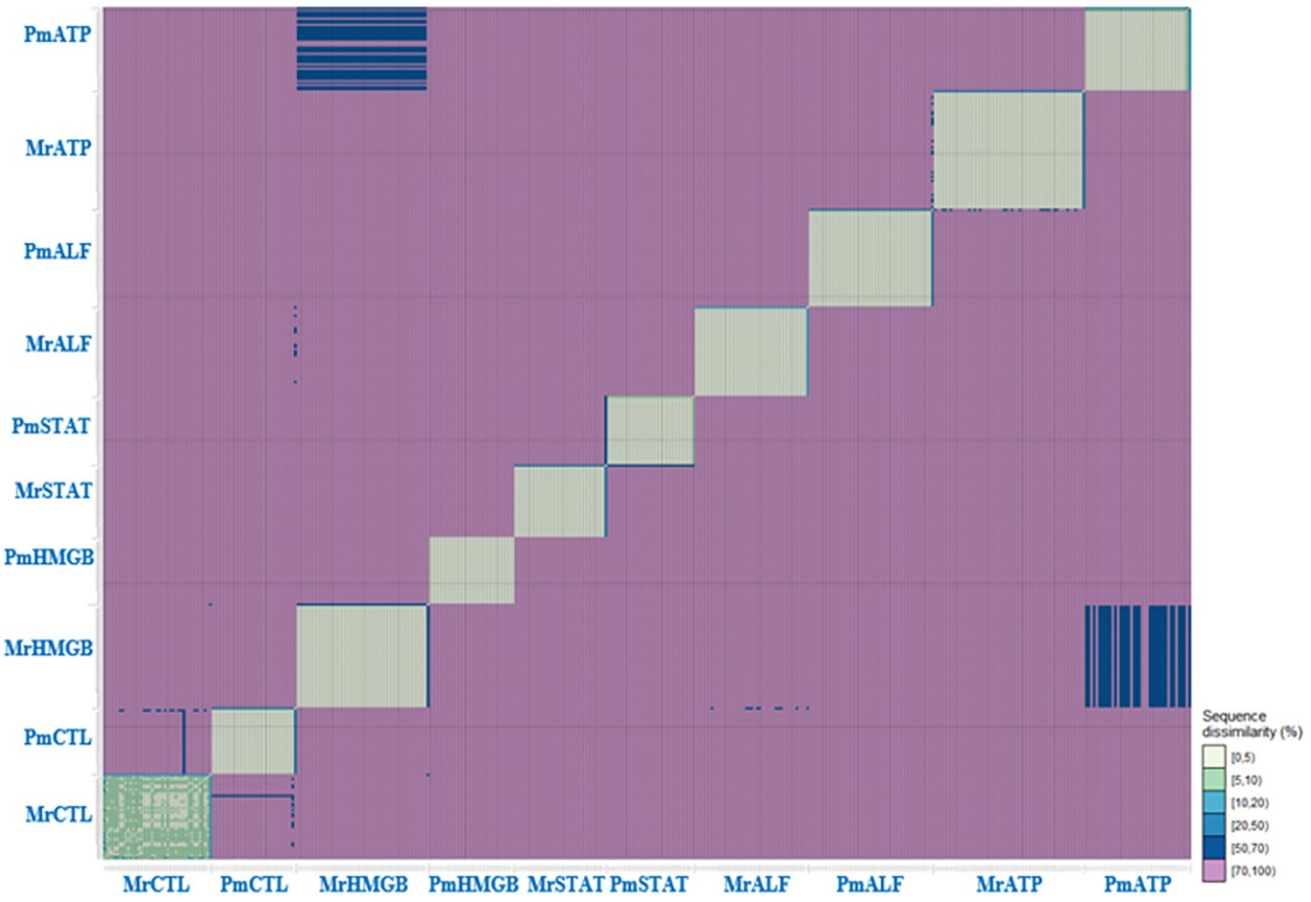
Display of genetic differentiation between different genes of total cDNA sequences. Mr: *M*. *rosenbergii*; Pm: *P*. *monodon*. Genes: C-type Lectin (CTL), HMGB, STAT, ALF3 (ALF), ATPase 8/6 (ATP).

**Fig 2:**
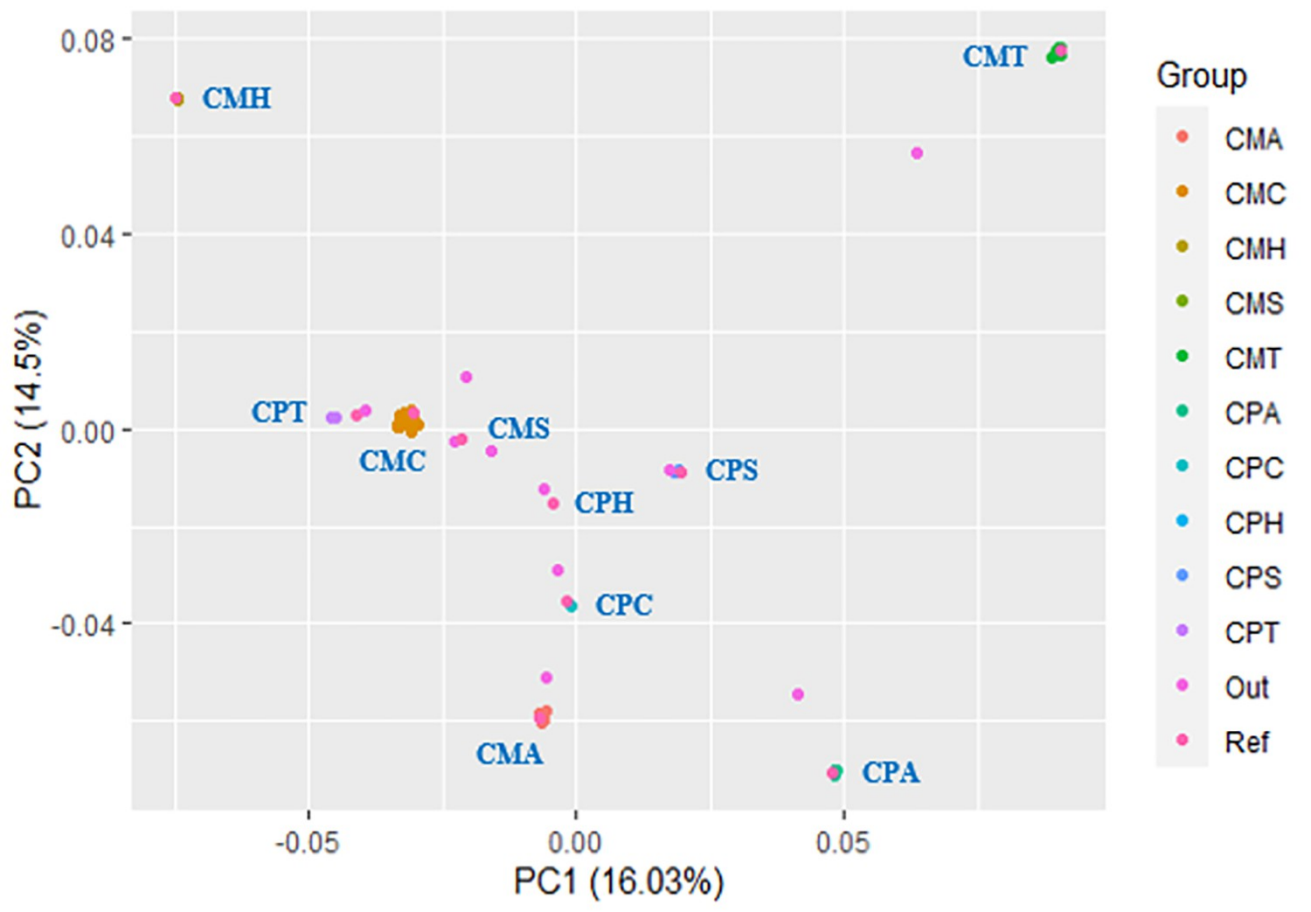
Principal component analysis (PCA) between different genes of total cDNA sequences. Mr: *M*. *rosenbergii*; Pm: *P*. *monodon*. CMA: Mr ALF3 cDNA, CMC: Mr C-type Lectin cDNA, CMH: Mr HMGB cDNA, CMS: Mr STAT cDNA, CMT: Mr ATPase 8/6 cDNA; CPA: Pm ALF3 cDNA, CPC: Pm C-type Lectin cDNA, CPH: Pm HMGB cDNA, CPS: Pm STAT cDNA, CPT: Pm ATPase 8/6 cDNA. Out: Outgroup; Ref: Reference.

Generally, *Penaeus monodon* and *Macrobrachium rosenbergii* gene sequences, which comprise five immune genes (C-type Lectin, HMGB, STAT, ALF3, and ATPase 8/6), were used for the genetic conservation or divergence analysis. A range of 30 to 50 sequences were utilized for each of the immune genes from both *P*. *monodon* and *M*. *rosenbergii*. The genetic conservation or divergence was initiated with AMOVA analysis to identify the degree of genetic differentiation as shown in (Table 1A–1C). From the AMOVA results, significant genetic differentiations (p<0.05) were determined between different genes, species-based combined gene sequences, and inter-species comparison within individual genes. The AMOVA results were supported by subsequent Population Matrix Differentiation and Population Pairwise FSTs analyses conducted. Based on the Population Matrix Differentiation analysis conducted (S2A Table), there were significant genetic differentiations (p<0.05) between different gene sequences of *P*. *monodon* and *M*. *rosenbergii*. This finding was validated through Population Pairwise FSTs analysis (S3 Table). Intriguingly, a subgroup comparison between *Vp*_AHPND_-infected and uninfected control *P*. *monodon* gene sequences (S2B Table) showed significant genetic differentiations (p<0.05) for the C-type Lectin, HMGB, and ALF3 genes. Among these genes, HMGB had the highest level of genetic differentiation between its subgroups. This finding was validated by the DNA divergence analysis as shown in S4 Table. The number of conserved and diverged sites (S5A & S5B Table) were counted at both nucleotide and translated amino acid levels in which major divergence was found at 5’ and 3’ regions. In addition, for individual gene sequence variations, *M*. *rosenbergii* C-type Lectin showed the highest divergence whereas *P*. *monodon* STAT showed the highest conservation. These significant genetic differentiations were supported and visualized by further analyses of genetic differentiation display (Fig 1) and PCA (Fig 2). Overall, from the evolutionary genetics analysis done, it can also be identified that the gene with the highest inter-species variance was STAT whereas the gene with the lowest inter-species variance was C-type Lectin.

**Table 1:** Measures of genetic differentiation based on analysis of molecular variance (AMOVA) approach using Arlequin software.

**(A) Genes**						
	**d.f.**	**Sum of Squares**	**Variance Component**	**Percentage of Variation (%)**	**Fixation Index (FST)**	**p** ^**a**^
Among populations	9	13348.094	38.19654	98.91	-	0.00000*
Within populations	380	160.060	0.42121	1.09	-	0.00000*
Total	389	13508.154	38.61775	100	0.98909	0.00000±0.00000*
**(B) Species-based Combined Gene Sequences (*M*. *rosenbergii* and *P*. *monodon*)**						
	**d.f.**	**Sum of Squares**	**Variance Component**	**Percentage of Variation (%)**	**Fixation Index (FST)**	**p** ^**a**^
Among populations	1	39363.397	1484.82728	98.05	-	0.00000*
Within populations	51	1503.603	29.48240	1.95	-	0.00000*
Total	52	40867.000	1514.30968	100	0.98053	0.00000±0.00000*
**(C) Inter-species Comparison within Individual Genes**						
		**Variance Component (Among populations)**		**Percentage of Variation (%)**	**Fixation Index (FCT)**	**p** ^**a**^
C-type Lectin		35.08103		95.87	0.95872	0.00000±0.00000*
HMGB		38.98945		99.97	0.99967	0.00000±0.00000*
STAT		38.00000		100	1.00000	0.00000±0.00000*
ALF3		33.47946		99.19	0.99189	0.00000±0.00000*
ATPase 8/6		42.22899		99.10	0.99101	0.00000±0.00000*

*Statistically significant (p<0.05)

^a^Probability of finding a more extreme variance component than the observed by chance alone after 1000 permutations.

Population 1: *M*. *rosenbergii* gene sequences

Population 2: *P*. *monodon* gene sequences

### 3.2 Selection forces

The same gene sequences from the genetic conservation or divergence analysis were further utilized for the analysis of selection forces. Neutrality tests, including Ewens-Watterson, Tajima’s D, and Fu’s Fs (Table 2), were successfully conducted to analyse the selection forces acting on different genes of *P*. *monodon* and *M*. *rosenbergii* together with different subgroups of *P*. *monodon*. The results revealed that strong purifying selection force was associated with immune genes of both shrimp species. Overall, *M*. *rosenbergii* had stronger purifying selection forces detected compared to *P*. *monodon* except for the *P*. *monodon* STAT gene which possessed the strongest purifying selection force. Balancing selection force was detected for *P*. *monodon* C-type Lectin gene uninfected control subgroup. Interestingly, *P*. *monodon* HMGB gene *Vp*_AHPND_-infected subgroup showed balancing selection force as well whereas its uninfected control subgroup showed purifying selection force. Besides that, directional selection forces were identified in *M*. *rosenbergii* HMGB and ATPase 8/6 genes along with *P*. *monodon* HMGB and STAT genes.

**Table 2:** Neutrality test using Arlequin software.

Sample Groups	Ewens-Watterson (F_OE_)	Tajima’s D (D)	Fu’s Fs (F_S_)
MrCTL_cDNA	-	0.61978	-5.69760
MrHMGB_cDNA	0.10227*	-2.00749**	-15.23859***
MrSTAT_cDNA	-0.00015	0.24862	-10.82544***
MrALF_cDNA	0.01642	-0.46800	-11.32571***
MrATP_cDNA	0.07382*	-1.10030	-5.94322
PmCTL_cDNA	-0.01593	1.10310	-2.66422
PmHMGB_cDNA	0.09438*	0.60207	2.49707
PmSTAT_cDNA	0*	-0.34324	-25.26160***
PmALF_cDNA	0.03019	0.12411	-5.61908***
PmATP_cDNA	-0.10251	0.40351	0.90836
PmCTL_N_cDNA	-0.03267	2.07464**	-1.58936
PmCTL_A_cDNA	-0.07507	0.36036	-0.35407
PmHMGB_N_cDNA	0.16658*	-2.23052**	2.14324
PmHMGB_A_cDNA	0.01840	2.08852**	2.00110
PmSTAT_N_cDNA	0.06574*	-0.36072	-9.67408***
PmSTAT_A_cDNA	-	-0.18171	-9.47945***
PmALF_N_cDNA	-0.01542	0.01260	-3.36184***
PmALF_A_cDNA	0.06957	-0.22667	-2.94485***
PmATP_N_cDNA	-0.10527	1.32709	0.96614
PmATP_A_cDNA	-0.09431	0.59123	0.61288

Mr: *M*. *rosenbergii*; Pm: *P*. *monodon*

Genes: C-type Lectin (CTL), HMGB, STAT, ALF3 (ALF), ATPase 8/6 (ATP)

N: Uninfected control subgroup; A: *Vp*_AHPND_-infected subgroup

Ewens-Watterson:

F_oe_<0 and P<0.025: Balancing selection

F_oe_>0 and P>0.975: Directional selection

(F_oe_: Deviate of homozygosity; Statistical Significance: P<0.025, >0.975)

Tajima’s D:

D = 0: Population evolving as per mutation-drift equilibrium. There is no evidence of selection.

D<0: A recent population expansion or purifying selection is suggested.

D>0: A recent population bottleneck or balancing selection is suggested.

(Statistical significance: D value of <-2 or >2; P<0.05)

Fu’s Fs:

F_S_< 0: A recent population expansion is suggested.

F_S_> 0: A recent population bottleneck is suggested.

(Statistical significance: P<0.02)

*Statistically significant (P<0.025 or >0.975)

**Statistically significant (P<0.05)

***Statistically significant (P<0.02)

### 3.3 Evolutionary divergence analyses

**Fig 3:**
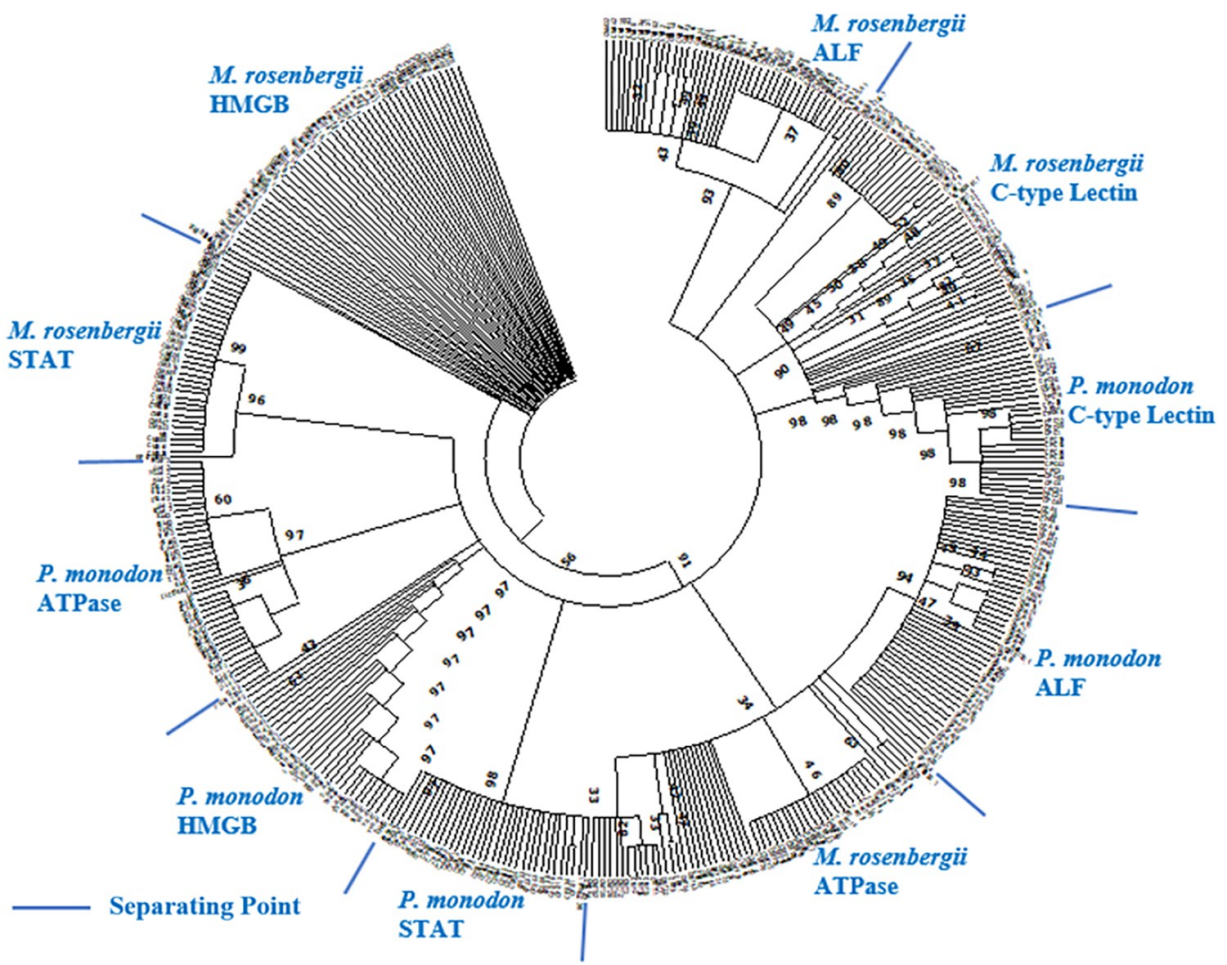
Maximum likelihood-based phylogenetic analysis generated using MEGA X software (Bootstrap: 1000) for total cDNA sequences. Sample groups: *M*. *rosenbergii* and *P*. *monodon*. Genes: C-type Lectin, HMGB, STAT, ALF3 (ALF), and ATPase 8/6 (ATPase).

**Fig 4:**
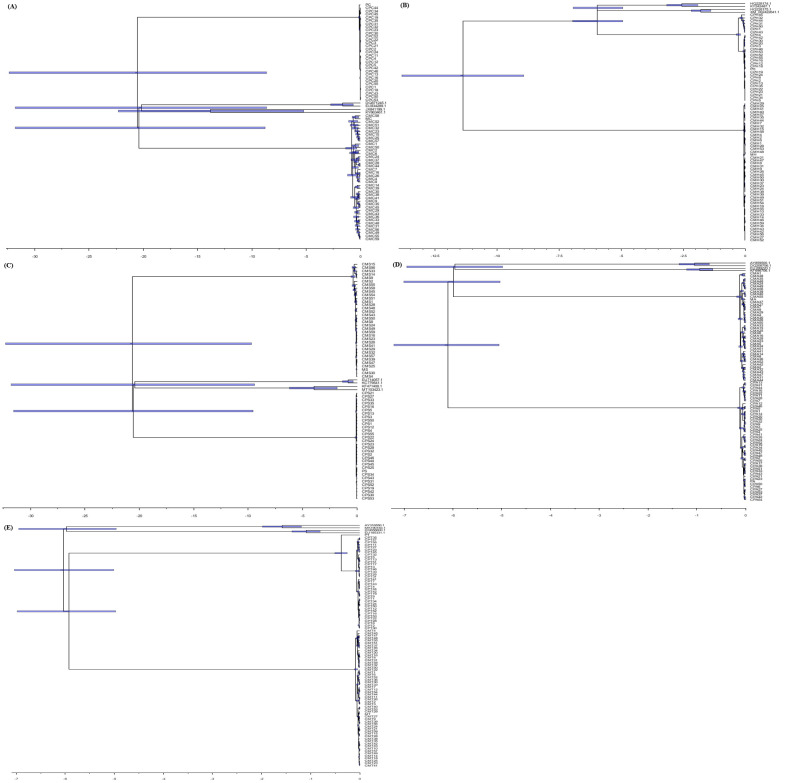
Bayesian molecular clock inference using BEAST2 software. A: C-type Lectin, B: HMGB, C: STAT, D: ALF3, E: ATPase 8/6. (Gamma site model; Relaxed clock log normal; Chain length: 10000000 or before infinity and ESS>200). [Models used: A (HKY+G), B (TrN+G), C (HKY+G), D (HKY+G), E (HKY+G)].

The total cDNA sequences used in the genetic and selection force analyses were analysed for evolutionary divergence through phylogenetic and Bayesian analyses. A maximum likelihood-based phylogenetic analysis was conducted using the total cDNA sequences as shown in Fig 3. From the phylogenetic analysis, it can be clearly observed that different gene sequences of *P*. *monodon* and *M*. *rosenbergii* were clustered accordingly. The gene sequences of C-type Lectin from the two shrimp species were grouped together in the closest distance followed by ALF3 gene sequences compared to other gene sequences. In addition, the genetic divergence times followed by event estimations of these immune genes between *P*. *monodon* and *M*. *rosenbergii* were determined through Bayesian Molecular Clock Dating method (Fig 4A–4E). The estimated divergence times obtained between *M*. *rosenbergii* and *P*. *monodon* immune genes were 438.6 million years ago (mya) (C-type Lectin), 1885.4 mya (HMGB), 432.6 mya (STAT), 448.1 mya (ALF3), and 426.4 mya (ATPase 8/6) respectively. The majority of the immune genes had divergence times in the Silurian period (443.7 mya to 416.0 mya) of the Paleozoic era (542 mya to 251 mya) except for the ALF3 gene that had time estimation in the Ordovician period (488.3 mya to 443.7 mya) and the HMGB gene that had time estimation in the Proterozoic era (2500 mya to 542 mya). Hence, a higher mutation rate was implied for the HMGB gene compared to the other genes which resulted in the very much earlier estimated divergence time in the Proterozoic era. The expected divergence time of the two shrimp species was indicated to be within the Paleozoic era as inferred from the estimated divergence times of the other genes obtained. The mentioned time periods were referred to a previously reported geological time scale [45, 46].

## 4 Discussion

### 4.1 Evolutionary genetic variations and selection forces

Evolutionary genetic variations were demonstrated through AMOVA analysis results which showed that the compared immune genes are significantly different from each other in the same shrimp species or between *Macrobrachium rosenbergii* and *Penaeus monodon*. The significant inter-species genetic differentiations inferred that *P*. *monodon* and *M*. *rosenbergii* are distinctly separated in terms of genetic evolution. This finding is supported by a previously constructed prophenoloxidase phylogeny tree in which *M*. *rosenbergii* did not cluster together with *P*. *monodon* or other Penaeidae shrimps [47].

Notably, *P*. *monodon* HMGB possessed the highest subgroup genetic divergence (between uninfected control and *Vp*_AHPND_-infected) followed by C-type Lectin and ALF3 compared to other genes. This observation was deduced to be caused by the occurrence of gene or transcript sequence alterations during AHPND infection for adaptation purpose due to the selection force exerted by the invading pathogen. A similar example would be the proposed host immune adaptation mechanisms triggered by HIV infection involving DNA methylation and alternative splicing, which then affected immune gene expression [48].

At individual species gene level, *M*. *rosenbergii* C-type Lectin had the highest level of variance whereas *P*. *monodon* STAT had the lowest level of variance. The individual species gene sequence variance is affected by the occurrence of Single Nucleotide Polymorphisms (SNPs) related to disease resistance [49]. The highest sequence variance shown in *M*. *rosenbergii* C-type Lectin gene was validated by the previously identified high sequence variations, additional domains, and diverse binding ability of the shrimp C-type Lectin due to its importance in immune recognition [50].

On the other hand, the lowest sequence variance shown in *P*. *monodon* STAT gene was postulated to be the effect of the highly conserved JAK/STAT pathway core components and functions in evolution [51, 52] as exemplified by the previously deduced grouping of the shrimp STAT into ancient STAT family [53]. These individual species gene variations were also associated with the reversed variance level observations at the inter-species level.

Genetic divergence is commonly found outside of conserved domains or translated regions. The highest degrees of divergence discovered in the 5’ and 3’ gene regions were inferred to be the effect of selection forces. This inference was supported by the identification of positive and negative selection forces that influenced the 5’ UTRs, 3’ UTRs, and cis-regulatory regions which then caused sequence variations in humans [54]. The vital function of 3’ UTRs in post-transcriptional regulation contributes to gene expression control and adaptive divergence [55].

The strong purifying selection force detected on the immune genes was postulated to be the main factor that caused the stronger immune response of *M*. *rosenbergii* in the recognition and elimination of invading pathogens. The *P*. *monodon* STAT gene with the strongest purifying selection force detected was also suggested to be crucial in the disease tolerance of the challenged *P*. *monodon* used in this study. Purifying selection is the most crucial evolutionary force of immune genes [25, 56–58] to achieve broad recognition of pathogens perhaps through conserved motifs [25]. Purifying selection was also inferred to be essential in the RNA sequence regulation for the maintenance of the correct RNA or protein functionalities in the innate immune system as shown by the previous discovery of its importance in the regulation of long dsRNA through RNA editing [59].

The *P*. *monodon* HMGB gene was postulated to be influenced by the balancing selection force formed during AHPND infection. Balancing selection plays vital role in the adaptive evolution across innate immune genes, particularly those involved in pathogen recognition. Immune genes showed molecular signatures of adaptation in response to various pathogens and associated selective pressures [60–62]. Besides that, directional selection was suggested to be an important evolutionary selection force acting on *M*. *rosenbergii* and *P*. *monodon* immune genes. The influence of directional selection on a Major Histocompatibility Class I marker in *Rana temporaria* was vital to achieve adaptation to Ranavirus [63]. A variable model of selection had also been determined for host-pathogen coevolution, which can be either balancing or directional selection for *Daphnia pulex* [61].

### 4.2 Evolutionary divergence and time estimation

The immune gene sequences of *M*. *rosenbergii* and *P*. *monodon* were compared and grouped accordingly in the phylogenetic analysis. Interestingly, *M*. *rosenbergii* and *P*. *monodon* C-type Lectin gene sequences were clustered closest to each other followed by ALF3 gene sequences compared to other gene sequences. This observation was postulated to be caused by the lowest level of divergence between *M*. *rosenbergii* and *P*. *monodon* C-type Lectin cDNA sequences among the compared gene sequences. C-type Lectin-like domains not only display recognition and functional versatility, but also possess highly conserved disulphide bridges associated with conserved hydrophobic and polar interactions [64]. The inter-species gene sequence conservation of C-type Lectin was supported by the previous demonstrations across different holometabolous insect species [65] and phylogenetically distinct viper species [66].

Besides that, similar inference of low ALF3 sequence divergence level between *M*. *rosenbergii* and *P*. *monodon* was also made to be accountable for the observed ALF3 gene sequences clustering. The inter-species gene sequence conservation of ALF3 was highlighted by the previously identified importance of conserved functional domain together with associated amino acid property and disulphide bond in maintaining the strong antibacterial activity of shrimp ALF [67]. Subsequently, the higher levels of inter-species evolutionary conservation shown in C-type Lectin and ALF3 genes compared to other immune genes were suggested to be the effect of their similar functional importance in the recognition and elimination of invading pathogens. This inference was validated by an example of the evolutionary conservation of a DExD/H-box helicase called DDX23 that plays vital role in the innate immune recognition of an RNA virus [68].

In addition, the estimated divergence times of C-type Lectin, STAT, ALF3, and ATPase 8/6 genes between *M*. *rosenbergii* and *P*. *monodon* were determined to be within the time range of 426.4 million years ago (mya) to 448.1 mya except for HMGB gene which was estimated at 1885.4 mya. These estimated divergence times were mostly grouped within the Paleozoic era (542 mya to 251 mya) [45, 46]. The main evolutionary event inferred to be responsible for these genetic divergences was the Cambrian Explosion. The Cambrian Explosion that happened around 520 mya to 515 mya caused the emergence of many different life forms on Earth, particularly those with hard body parts and eyes [69]. After the Cambrian animal diversification, the Ordovician and Silurian periods within the Paleozoic era were the crucial time periods of land colonization by arthropods and tetrapods [46].

The involvement of the oldest common ancestor of *M*. *rosenbergii* and *P*. *monodon* in the estimated genetic divergence was inferred as the oldest identified shrimp fossil can be traced back to the Devonian period (416 mya to 359.2 mya) [70]. Based on the fossil record, the occurrence of lineage divergence within the Penaeid shrimp superfamily, Penaeoidea was estimated to be around late Permian period (before 250 mya) [71]. This finding supported the estimated genetic divergence times between *M*. *rosenbergii* and *P*. *monodon* in the time periods before Permian period in this study. Furthermore, the deduced divergence time of the two shrimp species was shown to be within the Paleozoic era based on the estimated divergence times of the immune genes obtained. Intriguingly, a higher mutation rate was postulated for the HMGB gene evolution compared to the other genes which resulted in the very much earlier estimated divergence time in the Proterozoic era. The evolutionary mutation rates of different immune genes might be time dependent as exemplified by the previously demonstrated time dependency of human mitochondrial DNA mutation rate estimates [72]. Thus, the higher mutation rate of HMGB gene inferred might be caused by a unique evolutionary event that occurred in a particular time period.

## 5 Conclusion

In conclusion, significant genetic differentiations were identified among the immune-survival genes compared between *Macrobrachium rosenbergii* and *Penaeus monodon*, including C-type Lectin, HMGB, STAT, ALF3, and ATPase 8/6. For individual species genes, *M*. *rosenbergii* C-type Lectin showed the highest level of sequence divergence whereas *P*. *monodon* STAT showed the highest level of sequence conservation. For neutrality tests conducted, purifying selection was identified to be the main evolutionary force acting on the immune-survival genes of *M*. *rosenbergii* and *P*. *monodon* with overall stronger purifying selection force determined for *M*. *rosenbergii* immune-survival genes. The strongest purifying selection force was found on *P*. *monodon* STAT gene. Balancing selection was also determined to have occurred and acted on the *P*. *monodon* HMGB gene during AHPND infection. In addition, directional selection forces were identified for *M*. *rosenbergii* HMGB and ATPase 8/6 genes together with *P*. *monodon* HMGB and STAT genes. Based on the phylogenetic analysis, the C-type Lectin gene sequences were determined to be clustered closest between *M*. *rosenbergii* and *P*. *monodon* followed by ALF3 gene sequences compared to other genes. Furthermore, the Bayesian analysis successfully revealed estimated divergence times of C-type Lectin (438.6 mya), HMGB (1885.4 mya), STAT (432.6 mya), ALF3 (448.1 mya), and ATPase 8/6 (426.4 mya) genes between *M*. *rosenbergii* and *P*. *monodon*. The estimated divergence times were mostly within the Paleozoic era except for the unique estimated divergence time of HMGB in the Proterozoic era.

Evolutionary genetics analyses, including genetic differentiations, evolutionary selection forces, and estimation of divergence times were conducted for the immune-survival genes between *M*. *rosenbergii* and *P*. *monodon*. The important information uncovered from these evolutionary genetics analyses can bring deeper insight into shrimp genetic studies and potentially be referred by other researchers in similar field for experimental applications. Further evolutionary genetics analysis can also be conducted across many other shrimp species. The PCR primers designed in this study covering the conserved regions of immune-survival genes can be developed as shrimp health or immune activation diagnosis biomarkers. The developed biomarkers can then be utilized by shrimp farmers to minimize shrimp disease outbreaks. These biomarkers can assist in shrimp farm management and enhance precision aquaculture. The importance of biomarker application in shrimp aquaculture was emphasized by previous studies involving immune activation [73], temperature stress response [74], and environmental stress response [75]. The genetic information obtained can also assist in the development of stronger selective breeding programs to produce genetically enhanced shrimp lines with disease resistance or tolerance abilities.

## Supporting information

S1 Table List of primers designed and optimized for PCR amplification, (A) Primer information, (B) Primer sequences.
(DOCX)

S2 Table Genetic differentiation matrix of populations (p values) under population differentiation using Arlequin software: (A) Gene comparison (B) *P*. *monodon* uninfected control versus *Vp*_AHPND_-infected sub-group comparison.
(DOCX)

S3 Table Genetic differentiation matrix of populations calculated by FST and p values (shown in parenthesis) under population pairwise FSTs using Arlequin software. (DOCX)

S4 Table Measures of nucleotide diversity (π) and net nucleotide divergence (DNA divergence between populations) using DNASP software. (DOCX)

S5 Table Comparison of conserved and diverged sites in *M*. *rosenbergii* and *P*. *monodon* gene sequences at (A) Nucleotide and (B) Amino acid levels.
(DOCX)
