# Supplementary material for: Signature selection forces and evolutionary divergence of immune-survival genes compared between two important shrimp species
Source: PLoS One. 2023 Jan 12;18(1):e0280250. doi: 10.1371/journal.pone.0280250 (PMC9836293; doi:10.1371/journal.pone.0280250)
Supplement: S1 Table — List of primers designed and optimized for PCR amplification, (A) Primer information, (B) Primer sequences. (DOCX) [file pone.0280250.s001.docx]

**S1 Table**

**(A)**

| **Gene Sequence Information** | **Primer Name** | **Annealing Temperature (T_A_)** | **Targeted Region** |
| --- | --- | --- | --- |
| *M. rosenbergii* C-type Lectin (KX495215.1)  (Total: 1788 bp; Conserved domain: 263-637 bp) | MrCTL-1.2F & MrCtL-1R  MrCtL-2F & MrCtL-2R | 56.7 °C  56.7 °C | 64-983 bp  877-1744 bp |
| *P. monodon* C-type Lectin (DQ871244.1)  (Total: 1250 bp; Conserved domains: 173-544, 659-1024 bp) | C_PmCTL_F1 & C_PmCTL_R2  C_PmCTL_F2 & C_PmCTL_R2 | 56.7 °C  51.7 °C | 63-1025 bp  631-1025 bp |
| *M. rosenbergii* HMGB (Own Sequence)  (Total: 1484 bp; Conserved domains: 934-1131, 1198-1401 bp) | FUMrHG 1F & MrHG 2R  MrHG 2F & MrHG 2R | 56.7 °C  64.6 °C | 67-1393 bp  683-1393 bp |
| *P. monodon* HMGB (Own Sequence)  (Total: 1052 bp; Conserved domains: 171-374, 450-644 bp) | C_PmHG_F1 & C_PmHG_R1 | 56.7 °C | 20-988 bp |
| *M. rosenbergii* STAT (KT380661.1)  (Total: 2908 bp; Conserved domains: 151-525, 577-1158, 1162-1638, 1882-2232 bp) | FUMrST 2F & MrqST1R  MrqST1F & MrST_M 1R  MrST1F & MrST (3'-1R)  MrqST1F & MrqST1R | 52.1 °C  60.9 °C  52.1 °C  56.7 °C | 23-1065 bp  937-1672 bp  1573-2707 bp  937-1065 bp |
| *P. monodon* STAT (Own Sequence)  (Total: 2492 bp; Conserved domains: 138-518, 567-1148, 1152-1628, 1866-2216 bp) | FUPmST 2F & PMS 1R  PMS 2F & C_PmST_R3 | 56.7 °C  62.2 °C | 44-998 bp  920-2242 bp |
| *M. rosenbergii* ALF3 (KX610960.1)  (Total: 794 bp; Conserved domain: 180-470 bp) | MrALF-1F & MrALF-1R  MrALF-2F & MrALF-2R | 56.7 °C  64.3 °C | 41-705 bp  151-599 bp |
| *P. monodon* ALF3 (EF523559.1)  (Total: 476 bp; Conserved domain: 92-382 bp) | C_PmALF3_F01 & C_PmALF3_R1  C_PmALF3_F2 & C_PmALF3_R1 | 56.7 °C  56.7 °C | 3-426 bp  273-426 bp |
| *M. rosenbergii* ATPase 8/6 (Extracted from mitogenome, KY865098.1)  (Total: 828 bp; Conserved domain: 153-824 bp) | Mr_ATP8/6(F)_F & Mr_ATP8/6(F)_R  (Shafiee, 2018) | 60.0 °C | 1-812 bp |
| *P. monodon* ATPase 8/6 (Extracted from mitogenome,  NC_002184.1)  (Total: 828 bp; Conserved domain: 153-821 bp) | Pm_ATP8(R)_F & Pm_ATP6(R)_R | 56.7 °C | 108-621 bp |

**(B)**

| **Primer Name** | **Primer Sequence** |
| --- | --- |
| MrCTL-1.2F & MrCtL-1R | **MrCTL-1.2F:** 5'- TCGTCTGCTTGTGACCATTC -3'  **MrCTL-1R:**  5’- GGGCACCCAGATAGCTTTATAC -3’ |
| MrCtL-2F & MrCtL-2R | **MrCtL-2F:** 5’- CAAACTGCTATACTGTGCTTGT -3’  **MrCtL-2R:** 5’- TTTATCTCTTGAGCAGTAAGG C -3’ |
| C_PmCTL_F1 & C_PmCTL_R2 | **C_PmCTL_F1:**  5’- AACCTGTTCTCTGTTCGTAGG -3’  **C_PmCTL_R2:**  5’- CACACAGTGGGTAGAACACTGAA -3’ |
| C_PmCTL_F2 & C_PmCTL_R2 | **C_PmCTL_F2:** 5’- CGACCTGTTCGTGGAGGTG -3’  **C_PmCTL_R2:**  5’- CACACAGTGGGTAGAACACTGAA -3’ |
| FUMrHG 1F & MrHG 2R | **FUMrHG 1F:**  5'- GCTGTGGTGTAAGAAGATTATATGTC -3'  **MrHG 2R:** 5'- GCGTATGTCTGCCTATGCATTT -3' |
| MrHG 2F & MrHG 2R | **MrHG 2F:** 5'- ACTCAAGGGAGCCCATAACA -3'  **MrHG 2R:** 5'- GCGTATGTCTGCCTATGCATTT -3' |
| C_PmHG_F1 & C_PmHG_R1 | **C_PmHG_F1:**  5’- GTAAAGCGTCACTCGCTAAGA -3’  **C_PmHG_R1:**  5’- ACATGATATACGGTAACACGAGTC -3’ |
| FUMrST 2F & MrqST1R | **FUMrST 2F:**  5'- GCTTCATAAACCTCAAGGGATTTC -3'  **MrqST1R:** 5’- CTGATGTCGTTCACACTCTTT -3’ |
| MrqST1F & MrST_M 1R | **MrqST1F:** 5’- CAACAAATGGCTGGGAATGG -3’  **MrST_M 1R:**  5'- TTCTGGGACTGTGAAAGGTATG -3' |
| MrST1F & MrST (3'-1R) | **MrST1F:** 5’- GTCATTGTCCACGGTAATC -3’  **MrST (3'-1R)**: 5'- CCTAAGACACTATGGTTACACATCA -3' |
| MrqST1F & MrqST1R | **MrqST1F:** 5’- CAACAAATGGCTGGGAATGG -3’  **MrqST1R:** 5’- CTGATGTCGTTCACACTCTTT -3’ |
| FUPmST 2F & PMS 1R | **FUPmST 2F:** 5'- GCGAGTGCGTTGATTGACTC -3'  **PMS 1R:** 5’- CACACCATTCTTGTATCTGGTCT -3’ |
| PMS 2F & C_PmST_R3 | **PMS 2F:** 5’- TGGAAGAGAGACCAGCAGAT -3’  **C_PmST_R3:** 5’- TTAGTTGTGGGCTGTTGCTCTC -3’ |
| MrALF-1F & MrALF-1R | **MrALF-1F:** 5’- GTCTCGGAAACACGAGATCAG -3’  **MrALF-1R:** 5’- AGCGGGCTCTATAACAAGTTC -3’ |
| MrALF-2F & MrALF-2R | **MrALF 2F:** 5'- CGTCATCGTTACTTCCCACTT -3'  **MrALF 2R:**  5'- GATTGTGCCGTTGAGTAACATTC -3' |
| C_PmALF3_F01 & C_PmALF3_R1 | **C_PmALF3_F01:**  5'- TCCTAGTTTAGAAGATGCGTGTG -3'  **C_PmALF3_R1:**  5’- GCGGCTGAACACTGACAATTC -3’ |
| C_PmALF3_F2 & C_PmALF3_R1 | **C_PmALF3_F2:** 5’- CCAGCACACGCAGTCAGTC -3’ **C_PmALF3_R1:**  5’- GCGGCTGAACACTGACAATTC -3’ |
| Mr_ATP8/6(F)_F & Mr_ATP8/6(F)_R | **Mr_ATP8/6(F)_F:**  5'- ATCCCTCAAATAGCCCCCCTA -3'  **Mr_ATP8/6(F)_R:**  5'- AGCATAGAGGGTTCTTACGGC -3' |
| Pm_ATP8(R)_F & Pm_ATP6(R)_R | **Pm_ATP8(R)_F:**  5'- TGAGAAATCTTCATCTCAATTAC -3'  **Pm_ATP6(R)_R:**  5'- ATTACTGCTACAGCAGCTTCTAA -3' |
