# Supplementary material for: Signature selection forces and evolutionary divergence of immune-survival genes compared between two important shrimp species
Source: PLoS One. 2023 Jan 12;18(1):e0280250. doi: 10.1371/journal.pone.0280250 (PMC9836293; doi:10.1371/journal.pone.0280250)
Supplement: S2 Table — Genetic differentiation matrix of populations (p values) under population differentiation using Arlequin software: (A) Gene comparison (B) P. monodon uninfected control versus VpAHPND-infected sub-group comparison. (DOCX) [file pone.0280250.s002.docx]

**S2 Table**

**(A) Gene comparison**

|  | **MrCTL** | **MrHMGB** | **MrSTAT** | **MrALF** | **MrATP** |
| --- | --- | --- | --- | --- | --- |
| **PmCTL** | 0.00000±  0.0000* | 0.00000±  0.0000* | 0.00000±  0.0000* | 0.00000±  0.0000* | 0.00000±  0.0000* |
| **PmHMGB** | 0.00000±  0.0000* | 0.00000±  0.0000* | 0.00000±  0.0000* | 0.00000±  0.0000* | 0.00000±  0.0000* |
| **PmSTAT** | 0.00000±  0.0000* | 0.00000±  0.0000* | 0.00000±  0.0000* | 0.00000±  0.0000* | 0.00000±  0.0000* |
| **PmALF** | 0.00000±  0.0000* | 0.00000±  0.0000* | 0.00000±  0.0000* | 0.00000±  0.0000* | 0.00000±  0.0000* |
| **PmATP** | 0.00000±  0.0000* | 0.00000±  0.0000* | 0.00000±  0.0000* | 0.00000±  0.0000* | 0.00000±  0.0000* |

*Statistically significant (p<0.05); Markov chain (100000 steps)

Mr: *M. rosenbergii*; Pm: *P. monodon*

Genes: C-type Lectin (CTL), HMGB, STAT, ALF3 (ALF), ATPase 8/6 (ATP)

**(B) *P. monodon* uninfected control versus *Vp*_AHPND_-infected sub-group comparison**

|  | **PmCTL_N** | **PmHMGB_N** | **PmSTAT_N** | **PmALF_N** | **PmATP_N** |
| --- | --- | --- | --- | --- | --- |
| **PmCTL_A** | 0.01603±0.0019* | 0.00000±0.0000* | 0.00120±0.0004* | 0.00000±0.0000* | 0.00000±0.0000* |
| **PmHMGB_A** | 0.00000±0.0000* | 0.00240±0.0008* | 0.02938±0.0044* | 0.00000±0.0000* | 0.00000±0.0000* |
| **PmSTAT_A** | 0.00055±0.0001* | 0.00000±0.0000* | 0.47488±0.0103 | 0.00000±0.0000* | 0.00000±0.0000* |
| **PmALF_A** | 0.00000±0.0000* | 0.00000±0.0000* | 0.00000±0.0000* | 0.04660±0.0023* | 0.00000±0.0000* |
| **PmATP_A** | 0.00000±0.0000* | 0.00000±0.0000* | 0.00000±0.0000* | 0.00000±0.0000* | 0.50274±0.0027 |

*Statistically significant (p<0.05); Markov chain (100000 steps)

Pm: *P. monodon*

Genes: C-type Lectin (CTL), HMGB, STAT, ALF3 (ALF), ATPase 8/6 (ATP)

N: Uninfected control subgroup; A: *Vp*_AHPND_-infected subgroup
