# Supplementary material for: Signature selection forces and evolutionary divergence of immune-survival genes compared between two important shrimp species
Source: PLoS One. 2023 Jan 12;18(1):e0280250. doi: 10.1371/journal.pone.0280250 (PMC9836293; doi:10.1371/journal.pone.0280250)
Supplement: S3 Table — (DOCX) [file pone.0280250.s003.docx]

**S3 Table**

|  | **Combined Mr_cDNA** | **MrCTL** | **MrHMGB** | **MrSTAT** | **MrALF** | **MrATP** |
| --- | --- | --- | --- | --- | --- | --- |
| **Combined Pm_cDNA** | 0.98053 (0.00000)  * | - | - | - | - | - |
| **PmCTL** | - | 0.95872 (0.00000) * | - | - | - | - |
| **PmHMGB** | - | - | 0.99967 (0.00000) * | - | - | - |
| **PmSTAT** | - | - | - | 1.00000 (0.00000) * | - | - |
| **PmALF** | - | - | - | - | 0.99189 (0.00000) * | - |
| **PmATP** | - | - | - | - | - | 0.99101 (0.00000) * |

*Statistically significant (p<0.05) with 1000 permutations

Mr: *M. rosenbergii*; Pm: *P. monodon*

Genes: C-type Lectin (CTL), HMGB, STAT, ALF3 (ALF), ATPase 8/6 (ATP)
