# Supplementary material for: Signature selection forces and evolutionary divergence of immune-survival genes compared between two important shrimp species
Source: PLoS One. 2023 Jan 12;18(1):e0280250. doi: 10.1371/journal.pone.0280250 (PMC9836293; doi:10.1371/journal.pone.0280250)
Supplement: S4 Table — (DOCX) [file pone.0280250.s004.docx]

**S4 Table**

| **Samples** | **Nucleotide diversity (π)** ***#** | **Net nucleotide divergence, (Da)** |
| --- | --- | --- |
| Combined Mr_cDNA-Pm_cDNA | 0.39387 | 0.74021 |
| MrCTL_cDNA-PmCTL_cDNA | 0.39581 | 0.69636 |
| MrHMGB_cDNA-PmHMGB_cDNA | 0.37614 | 0.77096 |
| MrSTAT_cDNA-PmSTAT_cDNA | 0.37713 | 0.74132 |
| MrALF_cDNA-PmALF_cDNA | 0.36644 | 0.71397 |
| MrATP_cDNA-PmATP_cDNA | 0.36018 | 0.72892 |
| PmCTL_N_cDNA-PmCTL_A_cDNA | 0.00207 | 0.00041 |
| PmHMGB_N_cDNA-PmHMGB_A_cDNA | 0.01376 | 0.00347 |
| PmSTAT_N_cDNA-PmSTAT_A_cDNA | 0.00064 | 0.00004 |
| PmALF_N_cDNA-PmALF_A_cDNA | 0.00256 | 0.00018 |
| PmATP_N_cDNA-PmATP_A_cDNA | 0.00198 | -0.00002 |

* Estimated using Kimura two-parameter distance (Kimura, 1980); # Sites with gaps were completely excluded.

Mr: *M. rosenbergii*; Pm: *P. monodon*

Genes: C-type Lectin (CTL), HMGB, STAT, ALF3 (ALF), ATPase 8/6 (ATP)

N: Uninfected control subgroup; A: *Vp*_AHPND_-infected subgroup
