# Supplementary material for: Signature selection forces and evolutionary divergence of immune-survival genes compared between two important shrimp species
Source: PLoS One. 2023 Jan 12;18(1):e0280250. doi: 10.1371/journal.pone.0280250 (PMC9836293; doi:10.1371/journal.pone.0280250)
Supplement: S5 Table — Comparison of conserved and diverged sites in M. rosenbergii and P. monodon gene sequences at (A) Nucleotide and (B) Amino acid levels. (DOCX) [file pone.0280250.s005.docx]

**S5 Table**

**(A)**

| **Sample Groups** | **Number of Conserved Sites** | **Number of Diverged Sites (Variance; Singleton; Percentage of Divergence)** | **Total Number of Sites** | **Highest Region of Divergence** |
| --- | --- | --- | --- | --- |
| MrCTL | 1140 | 412; 48; 26.55% | 1552 | Overall |
| MrHMGB | 1050 | 26; 13; 2.42% | 1076 | Middle |
| MrSTAT | 2379 | 93; 12; 3.76% | 2472 | 3’ Region |
| MrALF | 568 | 21; 2; 3.57% | 589 | 5’ Region |
| MrATP | 718 | 27; 13; 3.62% | 745 | Overall |
| PmCTL | 853 | 5; 0; 0.58% | 858 | Middle |
| PmHMGB | 816 | 42; 5; 4.90% | 858 | 3’ Region |
| PmSTAT | 1877 | 12; 5; 0.64% | 1889 | 5’ Region and Middle |
| PmALF | 373 | 4; 1; 1.06% | 377 | 3’ Region |
| PmATP | 558 | 4; 0; 0.71% | 562 | 3’ Region |

Mr: *M. rosenbergii*; Pm: *P. monodon*

Genes: C-type Lectin (CTL), HMGB, STAT, ALF3 (ALF), ATPase 8/6 (ATP)

**(B)**

| **Sample Groups** | **Number of Conserved Sites** | **Number of Diverged Sites (Variance; Singleton; Percentage of Divergence)** | **Total Number of Sites** | **Highest Region of Divergence** |
| --- | --- | --- | --- | --- |
| MrCTL | 276 | 224; 23; 44.80% | 500 | Overall |
| MrHMGB | 323 | 18; 11; 5.28% | 341 | Middle and 3’ Region |
| MrSTAT | 754 | 58; 5; 7.14% | 812 | 3’ Region |
| MrALF | 178 | 13; 2; 6.81% | 191 | 5’ Region |
| MrATP | 202 | 21; 9; 9.42% | 223 | Middle and 3’ Region |
| PmCTL | 270 | 10; 5; 3.57% | 280 | Middle |
| PmHMGB | 255 | 20; 2; 7.27% | 275 | 3’ Region |
| PmSTAT | 590 | 7; 2; 1.17% | 597 | 5’ Region and Middle |
| PmALF | 120 | 2; 1; 1.64% | 122 | 3’ Region |
| PmATP | 180 | 3; 0; 1.64% | 183 | Middle |

Mr: *M. rosenbergii*; Pm: *P. monodon*

Genes: C-type Lectin (CTL), HMGB, STAT, ALF3 (ALF), ATPase 8/6 (ATP)
